# Instrumentation-Free Semiquantitative Immunoanalysis Using a Specially Patterned Lateral Flow Assay Device

**DOI:** 10.3390/bios10080087

**Published:** 2020-07-31

**Authors:** Kyung Won Lee, Ye Chan Yu, Hyeong Jin Chun, Yo Han Jang, Yong Duk Han, Hyun C. Yoon

**Affiliations:** Department of Molecular Science and Technology, Ajou University, Suwon 16499, Korea; ursus780@ajou.ac.kr (K.W.L.); yychan07@ajou.ac.kr (Y.C.Y.); moogoosla@ajou.ac.kr (H.J.C.); johkjj@ajou.ac.kr (Y.H.J.); aj23@ajou.ac.kr (Y.D.H.)

**Keywords:** lateral flow immunoassay, renal failure, microalbuminuria, instrument-free quantitative analysis

## Abstract

In traditional colorimetric lateral flow immunoassay (LFI) using gold nanoparticles (AuNPs) as a probe, additional optical transducers are required to quantify the signal intensity of the test line because it presents as a single red-colored line. In order to eliminate external equipment, the LFI signal should be quantifiable by the naked eye without the involvement of optical instruments. Given this objective, the single line test zone of conventional LFI was converted to several spots that formed herringbone patterns. When the sandwich immunoassay was performed on a newly developed semi-quantitative (SQ)-LFI system using AuNPs as an optical probe, the spots were colorized and the number of colored spots increased proportionally with the analyte concentration. By counting the number of colored spots, the analyte concentration can be easily estimated with the naked eye. To demonstrate the applicability of the SQ-LFI system in practical immunoanalysis, microalbumin, which is a diagnostic marker for renal failure, was analyzed using microalbumin-spiked artificial urine samples. Using the SQ-LFI system, the calibration results for artificial urine-based microalbumin were studied, ranging from 0 to 500 μg/mL, covering the required clinical detection range, and the limit of detection (LOD) value was calculated to be 15.5 μg/mL. Thus, the SQ-LFI system provides an avenue for the realization of an efficient quantification diagnostic device in resource-limited conditions.

## 1. Introduction

The development of an accurate and user-friendly diagnostic device is one of the most significant objectives in the clinical field. In the academic and industrial sectors, extensive research has proceeded to develop efficient diagnostic tools, such as point-of-care testing (POCT) biosensors [1,2,3,4,5,6,7]. To develop an efficient POCT-type immunosensor, researchers have focused on the lateral flow immunoassay (LFI) method, which is an immunochromatographic test using a nitrocellulose (NC) membrane as a substrate [8,9,10,11,12,13,14,15]. In traditional LFI, an analyte forms an immunocomplex with detection antibody-conjugated gold nanoparticles (Ab-AuNPs), then flows through the membrane and is captured on the test and control lines by capture antibodies that are immobilized on the lines. Since the operation of LFI is automatically accomplished by capillary force and the result can be verified with the naked eye, LFI has merits such as low cost of operation, easy handling, and operation without external instruments.

However, despite the merits of LFI, traditional colorimetric AuNP-based LFI is still used in limited tests that only require simple negative or positive results, such as pregnancy and sexually transmitted disease tests, because of the drawbacks associated with signal evaluation [16,17]. Typically, the result of an LFI test is presented as a single colored line on the strip that only indicates the existence of the target analyte. If quantification is desired, the color signal in the test line must be converted to optical density or color intensity values by an optical transducing device [18,19,20,21]. To overcome this weakness, barcode-type LFI strips that can easily quantify the analytes using LFI have been developed. However, this approach has limitations in the detailed quantitative analysis and requires an additional optical transducing device for further detailed quantitative analysis [22,23,24]. It also eliminates the inherent merits of LFI, such as its reasonable cost and equipment-free analysis. To develop a quantitative immunoassay using LFI while maintaining its inherent merits, the involvement of external transducing instruments should be avoided.

**Figure 1:**
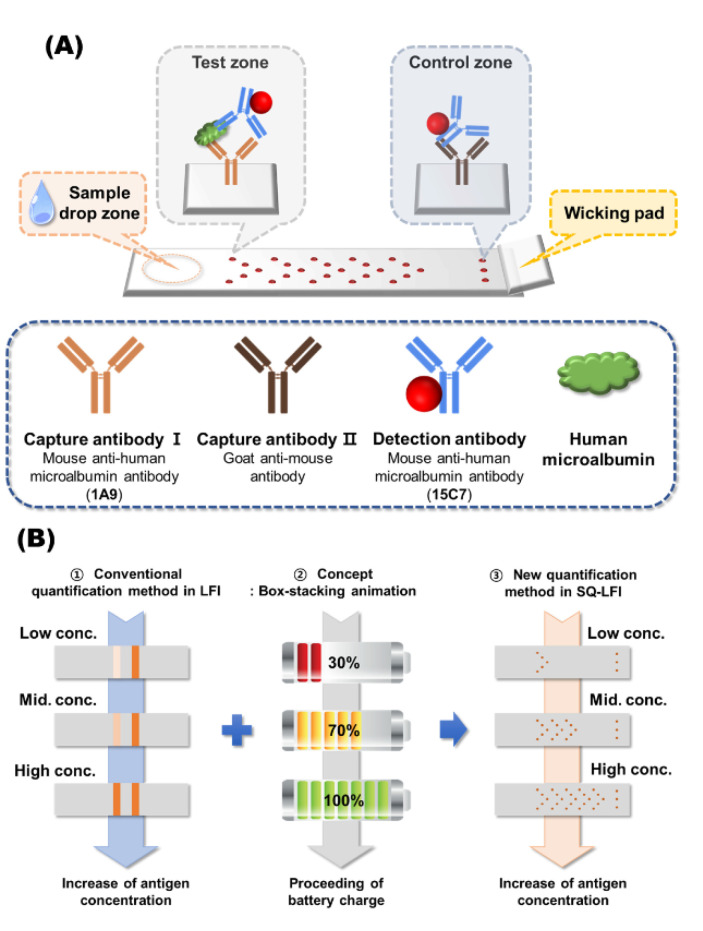
Schematic illustrations of the semi-quantitative lateral flow immunoassay (SQ-LFI) configuration and quantification strategy. (**A**) Configuration of the SQ-LFI system. (**B**) Quantification concepts of the SQ-LFI system.

With the aforementioned goal in mind, we developed a semi-quantitative LFI (SQ-LFI) method that does not require any external optical instrument to quantify the analytes (Figure 1A). For the simple quantitative analysis of analytes using SQ-LFI, the concept of battery charging animation was employed. Because the animation indicates the charging status of electronic devices as several small boxes, a user simply counts the number of stacked boxes to estimate the charging status (Figure 1B). By adopting this concept, we designed a new SQ-LFI system, in which the single test line of the conventional LFI was converted to several countable test spots. The developed SQ-LFI can provide information on the antigen concentration as several colored spots and, similar to concept animation, a user simply counts the number of colored spots to estimate the concentration of the antigen. In the sandwich-type immunoassay-based SQ-LFI, the numbers of colored spots that appear on the strip are proportional to the concentration of antigens in the samples. Because SQ-LFI proceeds automatically by capillary force and signal analysis can be conducted by the naked eye, quantitative analysis can be performed without any external instruments. In addition, despite the innovation of a new LFI test strip, the rest of the materials and procedures were the same as those used in conventional LFI. Therefore, the developed SQ-LFI can be produced using conventional methods with minimum procedural modifications.

To validate the applicability of SQ-LFI, a practical immunoanalysis for renal failure was conducted using artificial human urine samples. As a model biomarker of renal failure, we selected microalbumin, which is measured in urine as a specific indicator of glomerular filtration malfunction [25,26,27,28]. By analyzing microalbumin in the artificial urine with SQ-LFI, we acquired the calibration results for various microalbumin concentrations, which satisfied the clinically required detection ranges. In addition, we quantified the microalbumin concentration in artificial diabetes urine samples that contained a high concentration of glucose to include patients who suffer from renal failure as a complication of diabetes [29,30,31]. The SQ-LFI results met the clinical requirements with or without glucose in the artificial urine, which demonstrated the applicability of the SQ-LFI method in practical diagnostic scenarios.

In this study, we demonstrated that our SQ-LFI system allows for instrument-free immunoanalysis that can be applied to practical diagnoses and efficiently manufactured at conventional facilities. A detailed description of the sensing system and its detection performance is provided in the following sections.

## 2. Materials and Methods

### 2.1. Materials and Instruments

The NC membrane was obtained from mdi Membrane Technologies (Harrisburg, PA, USA). Gold chloride hydrate (HAuCl_4_), trisodium citrate, Triton X-100, Tween-20, L-arginine, polyvinyl alcohol (PVA, 9000–10,000 Da), glucose anhydrous, sucrose, albumin from human serum, and goat anti-mouse IgG antibody (capture antibody II) were purchased from Sigma-Aldrich (St. Louis, MO, USA). The Alexa Fluor^®^ 555-labeled rabbit anti-mouse antibody was obtained from Invitrogen (Carlsbad, CA, USA). Sodium chloride was purchased from Duchefa Biochemie (BH, Haarlem, Netherlands). Bovine serum albumin (BSA) was obtained from BioWorld (Dublin, OH, USA). Polyethylene glycol (PEG, 3400 Da) was obtained from Polyscience (Warrington, PA, USA). The mouse anti-human microalbumin antibody (clone number: 15C7, detection antibody) and mouse anti-human microalbumin antibody (clone number: 1A9, capture antibody I) were purchased from Hytest (Turku, Finland). Artificial urine was purchased from Pickering Laboratories (Mountain View, CA, USA). For the reaction solutions, 50 mM PBS buffer (pH 7.2), 0.1 M PBS buffer (pH 7.2), PBSB buffer (PBS buffer and 1% BSA), running buffer (1% BSA, 1% sucrose, 50 mM NaCl, 50 mM L-arginine, 0.2% PEG, 0.5% PVA, 0.2% Tx-100, and 0.2% Tween-20), and double distilled and deionized water (DDW) were prepared.

### 2.2. Preparation of Optical Probes, Including AuNPs and Ab-AuNPs

The 20 nm AuNPs were synthesized according to the procedure described in previous reports [32,33]. The size of the AuNPs was determined with an ultraviolet–visible (UV–vis) spectrophotometer by scanning from 300 to 800 nm (Figure S1A, Supporting information) [34]. Using the synthesized AuNPs, Ab-AuNPs were prepared according to the optimized protocols employed in our previous study. The final concentration of the detection antibody in the Ab-AuNP solution was 4 μg/mL (Figure S1B, Supporting information). The unreacted regions of the AuNP surface were blocked with BSA. The Ab-AuNPs were dispersed in 50 mM PBS containing 1% BSA at a maximum optical density of 1. The resulting Ab-AuNPs were stored at 4 °C until use.

### 2.3. Preparation of SQ-LFI Strips

To fabricate the SQ-LFI strips, the NC membrane was cut into rectangles (6 cm × 0.6 cm). To prepare the test zone and control zone, capture antibodies I and II were dotted on the NC membrane using the conventional metal nibs of dip pens. Using the capture antibody solution as ink, antibody spots were dotted on the desired region of the NC membrane (Figure S2A, Supporting information). The volume and concentration of the antibodies in each antibody droplet that was applied to the NC membrane were approximately 0.5 μL and 0.6 mg/mL, respectively. In the test zone, capture antibody I was dotted. To find the optimized test zone condition, different shapes of spot patterns and different concentrations of capture antibody I solution were examined. In the control zone, three spots were dotted using 1 mg/mL of capture antibody II solution, and this condition was used in all experiments. After antibody dotting, the antibody-dotted NC membrane (SQ-LFI strip) was dried at room temperature.

### 2.4. Operation of SQ-LFI Strips

The SQ-LFI system comprises three procedures, as depicted in Figure S2B (Supporting information). First, the prepared Ab-AuNP solution and target samples were mixed at a volume ratio of 99:1. The mixture was incubated for 15 min at room temperature to form antigen-antibody complexes between microalbumin and Ab-AuNPs via immunoaffinity binding. Second, 25 μL of the mixture were dropped onto the drop zone of the SQ-LFI strip. After 10 s, once the NC membrane had absorbed the mixture solution, 80 μL of running buffer were applied to the drop zone. On the strip, the running buffer solution flows through the membrane surface and pushes the pre-dropped mixture solutions from the drop zone onto the capture antibody-immobilized region. The flow rate of the fluids was enhanced by a wicking pad located on the edge of the strip, and the residual fluid accumulated in the wicking pad. The pre-formed microalbumin/Ab-AuNP complexes were captured and accumulated in the test zone by capture antibody I via sandwich-type immunoaffinity binding. Other molecules in the mixture that were not captured in the test zone flowed to the control zone and wicking pad by capillary flow. The unreacted Ab-AuNPs and uncaptured immunological complexes were captured and accumulated in the control zone by capture antibody II via immunoaffinity binding. After the immunoassay, all of the SQ-LFI results were presented as red-colored spots in the test zone and control zone. To record the test results from the SQ-LFI, each tested strip was registered as an image file using a smartphone camera.

Even though the resulting SQ-LFI images can be evaluated by the naked eye, the thresholds of the strip images were adjusted using ImageJ software (National Institutes of Health (NIH), Bethesda, MD, USA). The resulting adjusted images are provided in the manuscript, along with the original images to prevent arbitrary differences in the interpretation and acquisition of analytical results.

### 2.5. Examination of the Effect of Spot-Pattern Alterations on Color Development

In order to achieve a spot counting-based quantification strategy in LFIs, the effect of spot-pattern alterations on spot color development was examined, and the most efficient pattern was chosen. Using the previously described SQ-LFI strip fabrication method, three types of SQ-LFI strips with different test spot patterns were prepared. The patterns employed were as follows: a linear pattern comprising nine linearly arranged spots, a diagonal pattern comprising nine diagonally arranged spots, and a herringbone pattern comprising 15 herringbone arranged spots divided by three units. On the test zone spots of all strips, 1.0 mg/mL of capture antibody I solution was dotted. All control zone spots were prepared in the same pattern (three spots arranged in the transverse direction) with 1.0 mg/mL of capture antibody II solution. The SQ-LFI operating procedures were performed on the prepared strips with the microalbumin-spiked 0.1 M PBS sample containing 500 μg/mL of microalbumin.

### 2.6. Observation of Flows in the SQ-LFI

To demonstrate the split of the stream by the spot on the test zone, we observed the flow that passed the spots of the SQ-LFI strip. In this study, the fluorescence-labeled antibody was adopted as an optical probe instead of Ab-AuNPs because the fluorescence signal was much more intense than the color signal of the AuNPs. As a test zone on the SQ-LFI strip, nine spots were dotted linearly in the longitudinal direction with 1.0 mg/mL of capture antibody I solution. Then, 3 μL of 2 mg/mL Alexa Fluor^®^ 555-labeled rabbit anti-mouse antibody solution were dropped on the sample drop zone of the strip. Next, 80 μL of running buffer were applied to the sample drop zone to allow the fluorescence-labeled antibody solution to flow on the strip. The flow of fluorescent antibodies on the strip was observed using a fluorescence microscope. When the fluorescent antibody flow passed the capture antibody I spot on the strip, an image of the spot appearing on the fluorescence microscope was recorded and obtained.

### 2.7. Signal Optimization of the SQ-LFI

To optimize the signal of the SQ-LFI strip, the effect of the concentration of the capture antibody solution applied to the test zone on spot color development was investigated. The test set consisted of five units of herringbone patterns (a total of 25 spots). The test set was constructed with three different concentrations of capture antibody I solution. Test sets ①, ②, and ③ used 0.6, 0.8, and 1.0 mg/mL of capture antibody I solution, respectively. In each set, 0, 16, and 500 μg/mL of the microalbumin-spiked PBS sample were used to verify the optimum concentration of the capture antibody I solution for microalbumin analysis using the SQ-LFI.

### 2.8. Quantification of Microalbumin in Buffer Samples and Artificial Urine Samples

To demonstrate SQ-LFI performance in the quantitative analysis of a biomarker, various concentrations (0, 16, 31, 63, 125, 250, and 500 μg/mL) of microalbumin-spiked PBS samples were analyzed. The test strip of set ① in the previous experiment, which comprised five units of herringbone patterns dotted with 0.6 mg/mL of capture antibody I solution, was used. To certify the reproducibility of the SQ-LFI, this calibration test was performed three times. In addition, the practical feasibility of the SQ-LFI for applications in the microalbuminuria test was verified using artificial urine samples and artificial diabetes urine samples with various concentrations of microalbumin (0, 16, 31, 63, 125, 250, and 500 μg/mL). To certify the reproducibility of the SQ-LFI, this calibration test was performed five times. All of the resulting test strips were recorded as image files by a smartphone camera and reported with threshold-adjusted images to prevent arbitrary differences in the interpretation and acquisition of the analytical results. The obtained quantification results were registered in the microalbumin calibration curve.

## 3. Results and Discussion

### 3.1. Signaling Principle of the Developed SQ-LFI System

In traditional LFI, Ab-AuNPs are used as probes. These probes are captured on the test line of the LFI via sandwich-type immunoaffinity binding and are presented as a red-colored line. The captured probes are accumulated on the test line according to the concentration of the antigen in the sample. As a consequence, by analyzing the color intensity of the test line, the LFI can analyze not only the presence of the antigen but also quantify its concentration in the sample.

However, since the intensity of the color on the strip is difficult to analyze without optical transducing instruments, the quantification of antigens with conventional LFI requires additional instruments. To overcome this drawback, we introduced the concept of a box-stacking animation to the newly developed SQ-LFI. This concept animation visualizes the charging status as several countable boxes to easily identify the charging status of an electronic device by the naked eye. By applying this concept, the test line of a conventional LFI, which presents an uncountable color intensity signal, was converted into test spots of in an SQ-LFI that comprised several countable colored spots. The concentration of the antigen is visualized by the appearance of colored spots and can be quantified by counting the number of spots with the naked eye. As the antigen concentration increases, the number of colored spots is increased in the SQ-LFI, unlike the color intensity increase in the test line of conventional LFI. In the opposite conditions, when the antigen concentration decreases, the number of colored spots is decreased in the SQ-LFI rather than the color intensity decrease in the test line in conventional LFI. Based on this principle, the antigen concentrations in the sample could be analyzed by counting the colored spots with the naked eye, and the analytical performance of the SQ-LFI was verified by analyzing various concentrations of the target analyte microalbumin.

### 3.2. Effect of Spot-Pattern Alterations on the Signal Development of LFI

**Figure 2:**
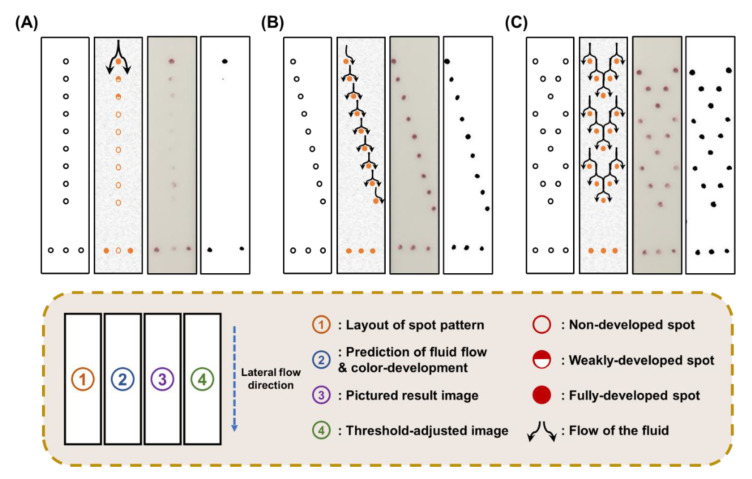
Examinations of various pattern types to select the most effective spot arrangement. (**A**) A linear pattern comprising nine linearly arranged spots. (**B**) A diagonal pattern comprising nine diagonally arranged spots. (**C**) A herringbone pattern comprising 15 spots arranged in a herringbone formation with five spots per unit.

The immunoassay result of a conventional LFI is provided to the user as a single colored line. Because a line can be regarded as the sum of numerous dots, we divided the line into several spots and distributed them on the test zone of the strip. The distributed spots allow for the quantitative analysis of an LFI with the naked eye. However, the accumulated immunocomplexes on the spots could affect the flow on the strip because of their large components, such as antigens, antibodies, and AuNPs [33]. The color development of the spot is not only determined by the concentration of antigens but also by the contact chance between the flow and spot. Therefore, the results of the immunoassay differed depending on the difference in the spot patterns. Based on this consideration, we examined the effect of spot pattern alterations on the color development of spots by using the three different spot patterns shown in Figure 2.

**Figure 3:**
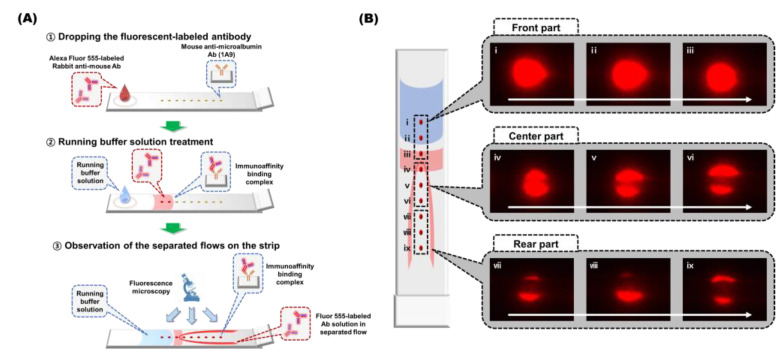
Schematic illustration of the flow observation procedure and resulting images of the flow around the developed spot. (**A**) Schematic illustration of the flow observation procedure. (**B**) Fluorescence images of the developed spots. The white arrow indicates the flow direction in the LFI.

**Figure 4:**
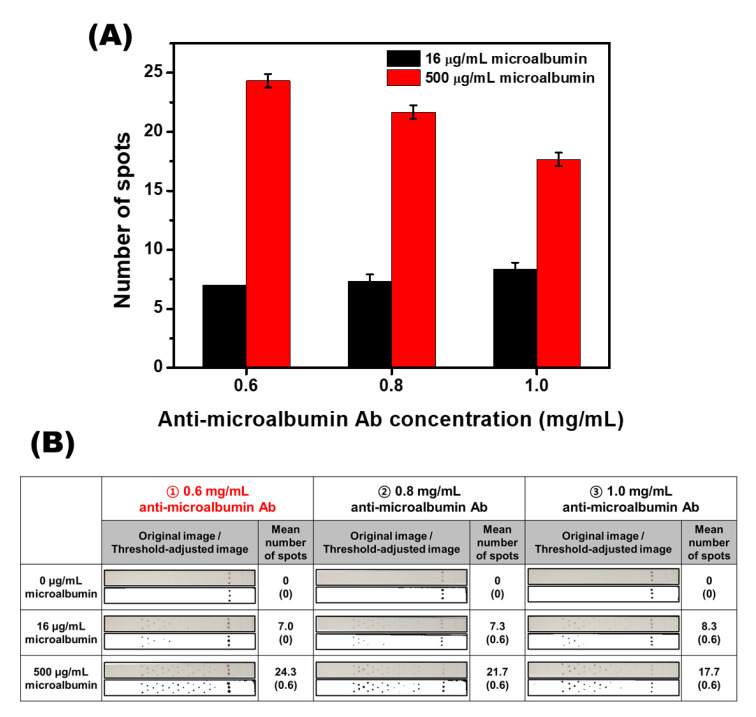
Optimization of capture antibody concentration in the test zone. (**A**) A graph representing the number of colored spots on strips using three different concentrations of capture antibody. (**B**) Original and threshold-adjusted images obtained from the immunoassay strip. The SD values are shown in parentheses.

A bundle of four images (①–④) is shown for each spot pattern test. The circled number ① indicates the spot pattern, ② suggests the prediction of flow, ③ shows the resulting image obtained by a smartphone camera, and ④ demonstrates the threshold-adjusted image to remove arbitrary interpretation. First, the pattern with nine linearly arranged spots (Figure 2A-①) was tested. In this pattern, it was expected that the immunocomplexes would accumulate at the foremost spot of the strip and act like a rock in a river to separate the flow (Figure 2A-②). After the immunoassay, only one fully developed spot was observed at the front, and the other eight subsequent spots were not colored or only slightly colored (Figure 2A-③ and -④). Interestingly, the center spot in the control zone at the bottom was not colored. In previous studies, López et al. and Yager et al. reported that the longitudinal flow based on capillary force was predominant in the transverse flow on the NC membrane strip [35,36,37]. The results in Figure 2A indicate that the first spot in the test zone, which is fully colored, acts as localized resistance and obstructs the flow of the reactant solution. We observed that the colored spot in the initial region on the SQ-LFI strip split the longitudinal flow. In addition, after the flow separation, the divided fluid was not combined as it proceeded to the end of the strip. This behavior was confirmed by observing that the central spot in the control zone was not colored. Based on these observations, we hypothesized that the large formed immunocomplexes, which comprised antigens, antibodies, and AuNPs, block the pores in the NC membrane and show resistance like a rock in a river. Naturally, the localized resistance in certain conduits changes the direction of flow from high-resistance regions to relatively low-resistance regions. In our experiment, the flow direction was changed at the first spot from the longitudinal direction to the transverse direction, resulting in a split. Because the longitudinal flow is predominant on an NC membrane, this changed flow direction was restored to the original direction immediately after flow disruption at the spot. Therefore, spots in the longitudinal direction should not be located at the same transverse coordinates so that the spots contact the flow, whose direction is split by upstream spots. Given this consideration, we modified the spot patterns to a diagonal line, as shown in Figure 2B-①. In this case, the transverse locations of all spots were different. Therefore, the divided flows from upstream spots could readily contact downstream spots, enhancing the color development of all spots (Figure 2B-②). As expected, all of the spots on the resulting test strip were fully colored (Figure 2B-③ and -④). These results indicate, via the projection of flow in Figure 2B-②, that the preceding spots and the latter spots should not be located at the same transverse coordinates when the spots are close together. However, even though the spots of the diagonal pattern present good color development, they cannot be directly used for quantitative immunoassays because the limited number of spots reduces the analytical performance. Considering this, we examined the herringbone pattern that contained a high density of signaling spots (Figure 2C-①). By the projection of the flow shown in Figure 2C-②, the herringbone pattern was expected to yield similar results to those of the diagonal pattern because the spot distributions in the transverse coordinates were analogous to those of the diagonal pattern. As expected, the herringbone pattern strips showed 15 fully colored spots. The colored spots in the herringbone pattern exhibited uniform color intensity even though the spot number was increased compared to the other spot patterns (Figure 2C-③ and -④). Based on these results, the herringbone-type spot pattern was selected for a spot counting-based quantification strategy in the newly developed SQ-LFI.

### 3.3. Flow Behavior Observation of the SQ-LFI Strip

To support our interpretation, including our prediction of flow behavior on the LFI strip, the flow around the developed spots on the SQ-LFI strips was observed using fluorescence microscopy. The movement of the Alexa Fluor^®^ 555–labeled anti-mouse antibody on the SQ-LFI strip is depicted in Figure 3A. We observed fluorescence changes in nine consecutive linear spots during the flow movement on the strip. Figure 3B shows the fluorescent images of nine linearly arranged spots.

The three spots located on the front part of the strip, shown in Figure 3B-i–iii, presented well-developed circular fluorescent spots, indicating that the expected immunoaffinity binding was complete. Next, in the center part comprising Figure 3B-iv–vi, a stream containing the fluorescent probe started to separate into two streams, as predicted in the previous section. After separation, the split stream flowed longitudinally through the strip without interruption. Therefore, the three spots in the rear part comprising Figure 3B-vii–ix presented the fluorescence signal on the longitudinal side edge of the spot. These results indicate that the colored spots generated by the formation of immunocomplexes induce imbalances in the flow rate on the strip. The capillary flow in the region where the membrane pores were occupied by immunocomplexes slowed down. In addition, since it seems that longitudinal flow is predominant over transverse flow in the strip, the split flow proceeded without the merging of tributaries. As a result, the fluid stream appeared to bypass the membrane areas where fluorescence signals had developed.

Interestingly, although the spot arrangement pattern was similar to the linear pattern examined in the previous section, the fluorescent dye-based examination of spot development was different from the AuNP-based examination. In the AuNP-based examination of the linear-patterned strip, the flow was separated at the front end of the spot. In contrast, a distinguishable flow separation was observed after spot number iv in the fluorescent dye-based test. The difference between these two examinations originated from the presence of AuNPs, which are rigid and larger than biomolecules. Because the accumulated AuNPs physically blocked the flow due to their material properties, flow separation was more easily observed than in the fluorescent dye-based examination. Considering the fluorescent dye-based flow result and physical properties of AuNPs, specially designed signaling spots, such as the herringbone pattern, have the advantage when constructing AuNP-based SQ-LFIs.

### 3.4. Signal Optimization of the Herringbone Pattern SQ-LFI

In the newly developed SQ-LFI technique, the color of the accumulated AuNPs in one test line was converted to several divided test spots. Therefore, for an accurate quantification assay using this principle, the factor of the divided spots was optimized. The major spot factors in the SQ-LFI were the number of spots and accumulation capacity of each spot. The number of spots was adjusted based on the target range of microalbumin (0 to 500 μg/mL) in this study. It was expected that the precise analysis of the desired range would not be possible with only 15 spots when used in the spot pattern test. Accordingly, 25 spots were applied to the test zone on the strip, which is the maximum number of spots that could be set on the strip with no interference from each other.

After the number of spots was determined, the accumulation capacity of each spot was considered. In the test zone, the accumulation capacity of the microalbumin/Ab-AuNP complexes was mainly determined by the amount of capture antibody I spotted on the strip. The more antibodies per spot, the more complexes that accumulated on each spot. Thus, the concentration of the antibody used as ink increased, and it was expected that fewer colored spots would be observed when measuring the highest concentration of the target. To optimize this condition, we tested three different concentrations of capture antibody I solution with 500 μg/mL of the microalbumin sample.

As shown in Figure 4A, the difference in the number of colored spots between the low target concentration sample and the high target concentration sample decreased according to the increased concentration of the capture antibody I solution. When the concentration of the capture antibody I solution was increased to 0.6, 0.8, and 1.0 mg/mL, the difference in the number of colored spots decreased to 17.3, 14.4, and 9.4, respectively. This tendency was predominantly affected by the decreasing number of colored spots in the samples with high concentrations of the analyte. The result indicates that the test set constructed with a high concentration of the capture antibody I solution consumed the microalbumin/Ab-AuNP complexes at a much earlier stage of the strip than the test set, which introduced a low concentration of the capture antibody I solution. Considering the clinical range of microalbumin (30 to 300 μg/mL), we selected the test set using 0.6 mg/mL of the capture antibody I solution as the optimized condition because the other sets differed too little between the low and high target concentration samples. In addition, in the blank images (0 μg/mL) shown in Figure 4B, the background signal by the nonspecific binding of biomolecules was completely eliminated with running buffer and showed no signal in all conditions. According to this result, colored spots presented by immunoanalysis in this strip are thought to be generated only by immunoaffinity binding without nonspecific binding and are expected to show high signal specificity.

### 3.5. Calibration Study with Microalbumin-Spiked PBS Samples

In the previous results, it was confirmed that the developed SQ-LFI could measure and distinguish the lowest and highest concentrations of microalbumin. To validate the feasibility of the developed SQ-LFI, a calibration study of microalbumin was performed prior to analysis of the real sample. For the calibration study, various concentrations of microalbumin-spiked PBS samples (0, 16, 31, 63, 125, 250, and 500 μg/mL) were used. The optimized SQ-LFI strip, which comprised five units of herringbone patterns dotted with 0.6 mg/mL of the capture antibody I solution, was fabricated and used in the test. To verify the reproducibility of the developed system for the detection of microalbumin, the same experiment was repeated under the same conditions at least three times.

**Figure 5:**
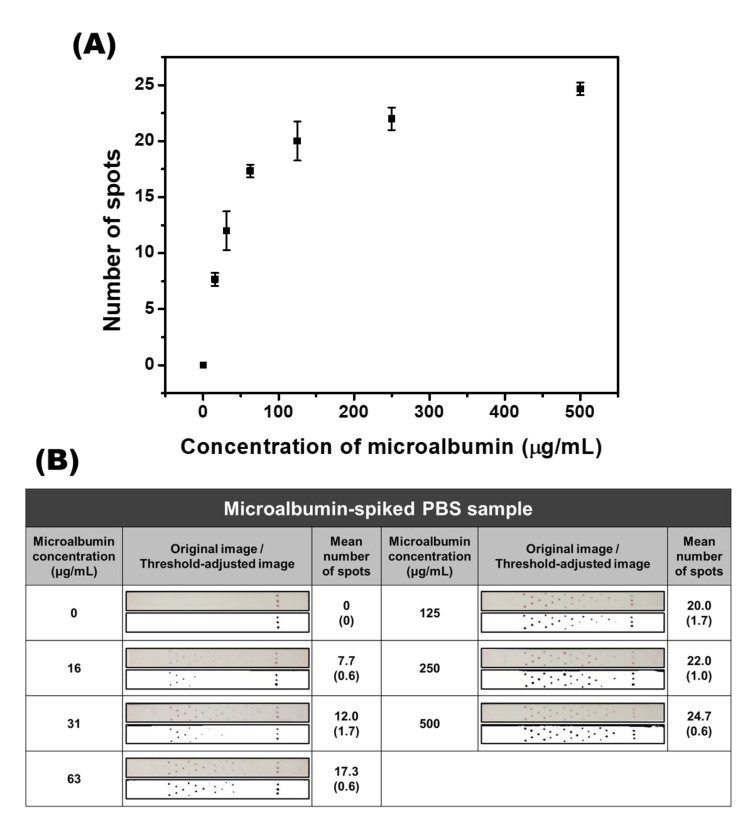
Quantitative analysis of microalbumin-spiked PBS samples. (**A**) The calibration curve of the various concentrations of microalbumin in PBS samples. (**B**) Original and threshold-adjusted images obtained from the immunoassay strip. The SD values are shown in parentheses.

The relationship between the concentration of microalbumin and the average number of colored spots is plotted as a calibration graph in Figure 5A. As shown on the graph, the low concentration region from 0 to 63 μg/mL shows good linearity, while the high concentration region from 125 to 500 μg/mL exhibits a saturation curve. The limit of detection (LOD) value was calculated as 13.5 μg/mL using the LOD calculation formulas represented below using the obtained calibration data.

$$SD\;of\;intercept=SE\;of\;intercept\times \sqrt{N}$$

LOD=3.3×(SD of intercept/slope)

Thus, the clinical range of microalbumin (from 30 μg/mL to 300 μg/mL) was covered. For further reliability, the original images and threshold-adjusted images are shown in Figure 5B. As displayed in the original images, the numbers of red-colored spots increased in proportion to the increasing concentration of microalbumin. For further accurate discrimination, the threshold was adjusted to the image of the strip. After the test, the numbers of colored spots on individual SQ-LFI strips were counted with the naked eye (no external instruments were employed for the quantifications), and the results from the tests in triplicate were averaged. Although the quantification procedures were performed without optical instruments, the acquired calibration results showed good sensitivity and reproducibility in a wide range of microalbumin concentrations.

### 3.6. Calibration Study with Standard Artificial Urine and Diabetes Artificial Urine Samples

Based on the reported results, it was verified that the developed SQ-LFI system could be used for the quantitative analysis of microalbumin under buffer conditions. However, in the development of a diagnostic system or device, the demonstration of its practical applicability in the analysis of real human samples is a very significant step. Therefore, to demonstrate the applicability of the developed system to human samples, an artificial urine solution containing various components (potassium dihydrogen orthophosphate, ammonium chloride, sodium chloride, sodium sulfite, disodium hydrogen orthophosphate, creatinine, and urea) was prepared and used. In this solution, microalbumin was spiked with seven different concentrations (0, 16, 31, 63, 125, 250, and 500 μg/mL), and these samples were analyzed with the developed SQ-LFI. The detection of microalbumin using the SQ-LFI was repeated under the same conditions at least five times to validate the reproducibility of the sensor.

**Figure 6:**
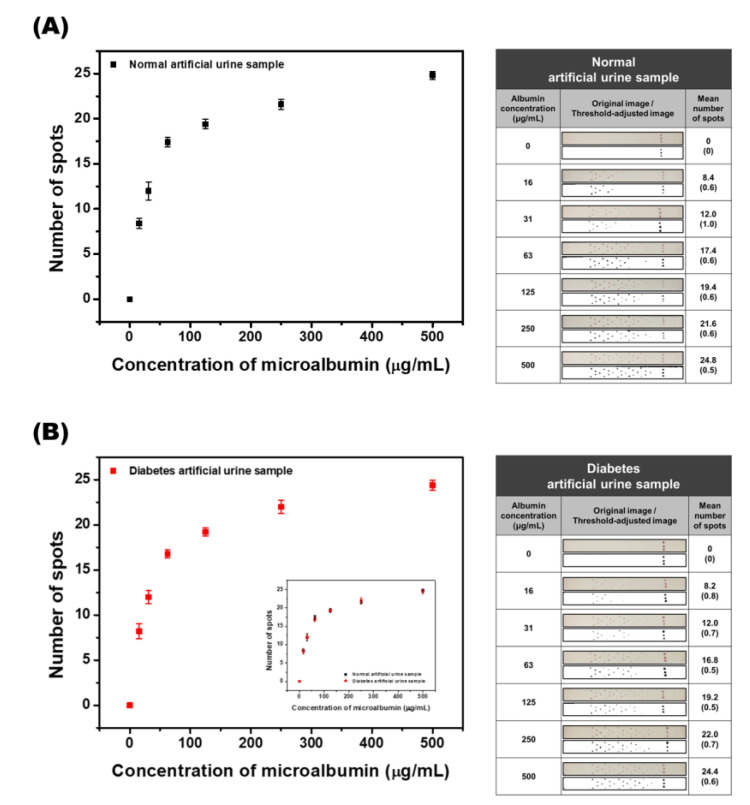
Quantitative analysis of microalbumin-spiked artificial urine and diabetes urine samples. (**A**) The calibration curve of microalbumin at various concentrations in the artificial urine samples. Original and threshold-adjusted images of the strip. (**B**) The calibration curve of microalbumin at various concentrations in the artificial diabetes urine samples. Original and threshold-adjusted images of the strip. The two calibration curves are overlapped in the inset graph. The SD values are shown in parentheses.

As shown in Figure 6A, the results of the calibration study with artificial urine samples presented a shape similar to that of the PBS sample. To increase the reliability of the results, not only the graph but also the images of the strip are reported. The number of colored spots increased in accordance with the increase in the concentration of microalbumin when the developed SQ-LFI was used as an analyzer. In the low concentration region of 0 to 63 μg/mL, the number of colored spots linearly increased. Since the graph presents an LOD value of 15.5 μg/mL and a sharp linear slope in the low concentration region, which contains the 30 μg/mL clinical cutoff value for normo-albuminuria and microalbuminuria, the developed biosensor could precisely distinguish normal and renal failure patients. The calibration curve in the high concentration region, from 125 to 500 μg/mL, exhibits a leveling off of the spot number counted. This region contains the 300 μg/mL clinical cutoff value of microalbuminuria and macroalbuminuria, which determines whether the disease is treated as mild or severe renal failure. The slope of the calibration curve is gentle in the high concentration region, but the differences between the spots of each concentration are distinguishable. The calculated linear graph is y = 0.01409x + 17.81463 in the high concentration region. According to this formula, the cutoff value of severe renal failure (300 µg/mL) presented 22.0 colored spots. So, in case the number of observed spots is under 22, the renal failure stage of the patient can be considered to be mild. In case the number of observed spots is 22, however, the patient can be treated as a high-risk patient for severe renal failure and additional tests for further verification are needed. On the other hand, patient samples exhibiting 23 to 25 colored spots can be diagnosed with severe renal failure. The results of analyzing the artificial urine samples demonstrated that the developed SQ-LFI could be used as a quantitative microalbumin biosensor with the ability to cover the clinical range.

In addition, since many cases of renal failure are reported as a complication of diabetes, it is likely that there are patients with simultaneous renal failure and diabetes. Therefore, to include these patients, the developed SQ-LFI should have the ability to sense microalbumin even if the urine sample contains a high concentration of glucose. An artificial urine sample containing 500 μg/mL of glucose was prepared and used as a diabetes artificial urine sample. The result of the calibration curve is shown in Figure 6B. The calibration graph presents a similar shape to the artificial urine sample graph. The inset graph shows the overlap of the two calibration curves. The result indicates that the analytical performance of the developed SQ-LFI was not hampered by the compositional change in the sample solution.

As seen in this study, the coverage of the developed SQ-LFI covers the clinical range of microalbumin, and the degree of renal failure can be determined based on each cutoff value. In addition, samples with high concentrations of glucose resulted in the same calibration curves as those without glucose; thus, the same performance can be demonstrated for patients with renal failure regardless of whether or not they also have diabetes.

## 4. Conclusions

We have developed an instrument-free SQ-LFI system with simple modifications to a conventional LFI strip. By converting the test line of the conventional LFI strip into bundles of spots arranged in a herringbone pattern, quantitative information on the target molecule can be provided as the number of colored spots on the test strip instead of color intensity. This approach allows for SQ immunoanalysis of the target antigen based on the counting of colored spots using the naked eye. Because SQ-LFI does not require any additional optical instruments, the developed immunosensing method can be used under conditions where electricity is not readily available. Furthermore, the analytical performance of SQ-LFI fulfills the clinical range requirements for renal failure, even in artificial urine and diabetes urine samples. Because the materials and fabrication procedures are the same as those used for conventional LFI, SQ-LFI can also be efficiently implemented using conventional facilities for LFI manufacturing.

## Supplementary Materials

The following are available online at https://www.mdpi.com/2079-6374/10/8/87/s1. Figure S1: Absorbance spectrum and image of the synthesized AuNP solution., Figure S2: Schematic diagrams of the SQ-LFI fabrication and operation.
