# Supplementary material for: Instrumentation-Free Semiquantitative Immunoanalysis Using a Specially Patterned Lateral Flow Assay Device
Source: Biosensors (Basel). 2020 Jul 31;10(8):87. doi: 10.3390/bios10080087 (PMC7460358; doi:10.3390/bios10080087)

Supplementary information

Instrumentation-free semiquantitative immunoanalysis using a specially patterned lateral flow assay device

Kyung Won Lee, Ye Chan Yu, Hyeong Jin Chun, Yo Han Jang, Yong Duk Han, and Hyun C. Yoon*

Department of Molecular Science and Technology, Ajou University, Suwon 16499, South Korea

* Author to whom correspondence should be addressed:

E-mail: hcyoon@ajou.ac.kr

Figure S1. (A) Absorbance spectrum of the synthesized AuNP solution. (B) Image and absorbance spectrum of the AuNP solution containing various concentrations of antibodies and a high concentration of NaCl.

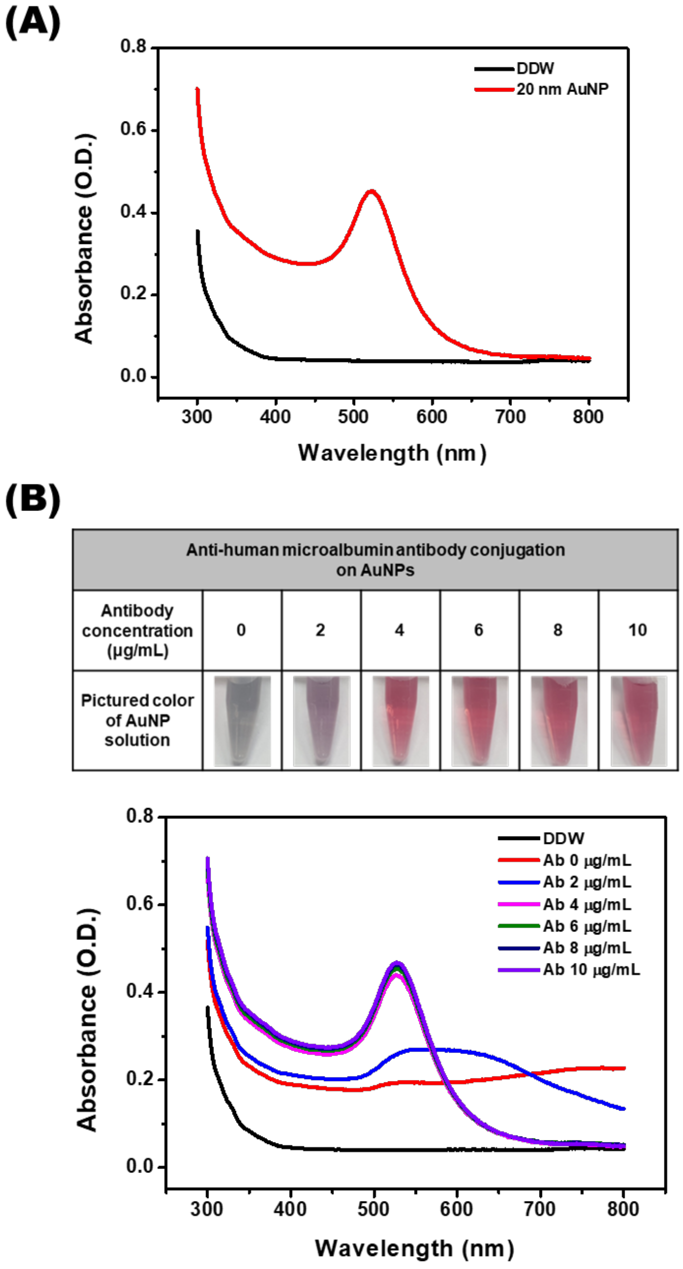

Figure S2. Schematic diagrams of the SQ-LFI fabrication and operation. (A) Preparation of the SQ-LFI strip. (B) Operation procedures of the SQ-LFI.

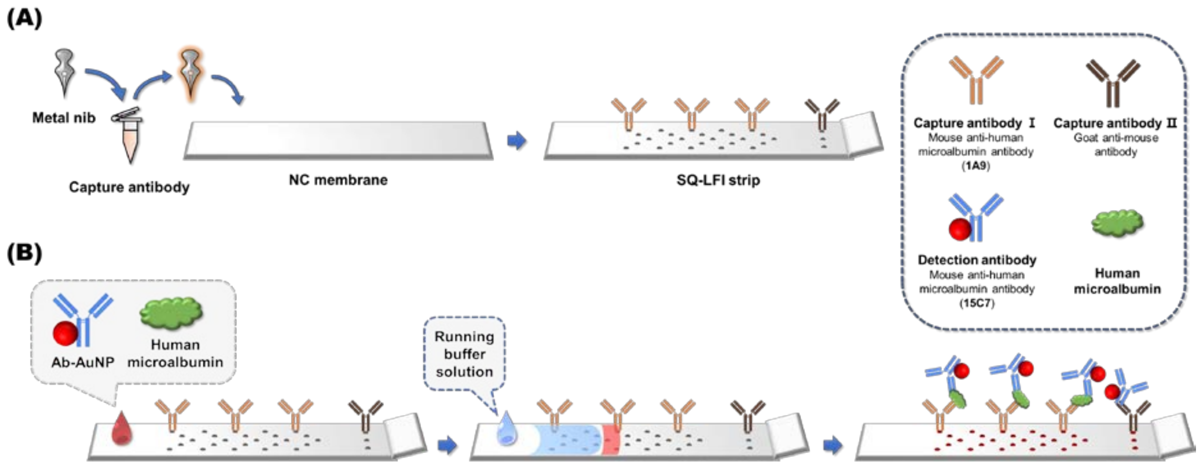

Supplement: Supplementary file 1 [file biosensors-10-00087-s001.pdf]
